# Evaluation of the Influence of Freeze‐Dried Gooseberry Addition on the Quality Characteristics of Model Pork Pâtés

**DOI:** 10.1155/ijfo/2041244

**Published:** 2026-11-14

**Authors:** Paulina Kęska, Kinga Zmorczyńska, Joanna Stadnik

**Affiliations:** ^1^ Department of Animal Food Technology Faculty of Food Science and Biotechnology University of Life Sciences in Lublin Lublin Poland up.lublin.pl

**Keywords:** antioxidants, food quality, freeze-dried gooseberry

## Abstract

The use of plant‐derived antioxidant ingredients in meat products has gained increasing attention because of their potential effects on oxidative processes and their possible application as alternatives to synthetic additives. This study evaluated the effect of freeze‐dried gooseberry (*Ribes uva-crispa* L.) on the physicochemical properties, antioxidant status, color, and texture of model pork pâtés during refrigerated storage. Pork pâtés containing 0.5%, 1%, and 2% freeze‐dried gooseberry were produced and stored at 4°C. Analyses were conducted on Days 1, 7, 14, 21, and 28. The addition of gooseberry significantly reduced the pH of the pâtés but did not affect water activity. Lightness (*L* ^*^) decreased with increasing levels of gooseberry. Gooseberry enrichment significantly increased ABTS radical scavenging activity, reaching 84.7% in the sample containing 2% gooseberry on day 14. TBARS values ranged from 1.30 to 2.30 mg MDA/kg during storage, with significant storage‐related changes observed within some formulations, while no significant differences between treatments were detected at any of the individual storage times. Freeze‐dried gooseberry enhanced the antioxidant capacity of pork pâté extracts without major adverse effects on the physicochemical and textural properties evaluated. The addition of up to 2% freeze‐dried gooseberry may therefore be considered a potential natural ingredient for increasing the measurable antioxidant potential of pork pâté without major adverse effects on the physicochemical and textural properties evaluated in this study.

## 1. Introduction

In recent years, increasing consumer awareness of the relationship between diet and health has stimulated the development of food products with improved nutritional and functional properties. In the meat industry, particular attention has been paid to the incorporation of plant‐derived ingredients rich in biologically active compounds. Although the use of plant materials in meat processing is not a new concept, their technological role has evolved considerably. Traditionally, plant components such as leaves, roots, stems, or fruits have been used mainly as spices to enhance flavor and aroma. However, these raw materials are also recognized as valuable sources of phytochemicals, including polyphenols, vitamins, and other compounds exhibiting antioxidant activity [1–3]. The addition of plant‐based ingredients to meat products may influence not only their sensory characteristics but also several technological and physicochemical properties. Parameters such as color, texture, acidity, and water activity may be modified by the presence of plant materials. These factors are closely related to the microbiological stability, shelf life, and overall quality of meat products [4, 5]. For this reason, the use of plant‐derived additives requires careful technological consideration, as their effects may vary depending on the type of raw material, concentration, and processing conditions. Moreover, the effectiveness of plant‐derived ingredients cannot be considered independently of the food matrix and the physical form in which the plant material is incorporated, as these factors may determine the availability and interactions of bioactive compounds within the product.

One of the main factors responsible for the deterioration of meat product quality during storage is oxidative degradation of meat components, particularly lipids and proteins. These reactions are initiated by reactive oxygen species (ROS), commonly referred to as free radicals, and may be intensified by environmental factors such as oxygen availability, light exposure, and storage conditions. As a consequence, oxidative processes may lead to undesirable changes in flavor, aroma, color, and texture, as well as to a reduction in the nutritional value and shelf life of meat products [6–10]. For this reason, considerable research has focused on the application of natural antioxidants in meat systems, especially those derived from plant sources [10]. Fruits are widely recognized as important sources of antioxidant compounds. European gooseberry (*Ribes uva-crispa* L.) contains phenolic compounds and ascorbic acid, which contribute substantially to its antioxidant activity [8]. The antioxidant potential of *Ribes uva-crispa* has been confirmed for different cultivars, although considerable variation in the concentration and profile of individual bioactive compounds has been reported [8, 9]. Because of this composition, European gooseberry represents a potentially valuable source of natural antioxidants for food applications. However, the effectiveness of plant‐derived antioxidants in complex food matrices, such as meat products, may depend on multiple factors, including their concentration, chemical interactions with meat components, processing conditions, and storage time. Consequently, antioxidant activity demonstrated in the fruit itself or in its extracts does not necessarily correspond directly to antioxidant effectiveness after incorporation into a processed meat matrix.

Previous studies evaluating gooseberry‐derived ingredients in meat products have reported beneficial effects on oxidative stability, color, texture, and storage quality [10–17]. However, these studies have largely involved botanically distinct fruits commonly referred to as “gooseberry”, particularly Indian gooseberry (amla; *Phyllanthus emblica* L., syn. *Emblica officinalis*) [11, 12] and Ceylon gooseberry [9], as well as gooseberry preparations used in poultry and mutton products [13–17]. Therefore, evidence obtained for these raw materials cannot be directly extrapolated to European gooseberry (*Ribes uva-crispa* L.), owing to differences in botanical origin and phytochemical composition. This distinction is particularly relevant when considering their functionality as sources of antioxidants in complex food matrices.

Despite these well‐documented benefits, the available literature remains limited in several important aspects. Previous studies have primarily investigated gooseberry‐derived ingredients in products such as nuggets, meatballs, patties, mutton rolls, and restructured meat products [10–17], whereas information concerning their application in highly comminuted and heat‐processed meat matrices, such as pork pâté, remains limited. Such products constitute complex multiphase matrices containing substantial lipid and protein fractions, in which comminution increases contact between lipids, pro‐oxidant components, and oxygen, while thermal processing may modify proteins, endogenous antioxidant systems, and the accessibility of oxidation substrates [6, 7]. Consequently, antioxidant effects observed in other meat matrices cannot necessarily be assumed to occur to the same extent in pork pâté.

Another important distinction concerns the physical form of the plant ingredient. Previous studies on gooseberry in meat products have predominantly employed fresh pulp, paste, conventional fruit powders, extracts, or combinations of fruit‐derived ingredients [12–17]. Freeze‐drying represents a technologically different approach. Freeze‐drying (lyophilisation) is considered one of the most effective methods for retaining the nutritional and functional properties of plant materials, as it allows water removal under low‐temperature conditions, thereby minimizing degradation of sensitive compounds and maintaining high antioxidant activity [18, 19]. The suitability of this approach for European gooseberry has also been demonstrated by Hussain et al. [9], who identified phenolic compounds and substantial antioxidant activity in extracts obtained from freeze‐dried and powdered red and green *Ribes uva-crispa* fruits. However, preservation of thermolabile compounds is not the only potential advantage relevant to meat products. In contrast to fresh pulp or paste, freeze‐dried fruit represents a low‐moisture and concentrated plant matrix. Its incorporation may therefore introduce bioactive compounds without simultaneously introducing the relatively high amount of water associated with fresh fruit material. Moreover, freeze‐drying followed by size reduction may affect the concentration, dispersion, and accessibility of phytochemicals and their interactions with the aqueous, lipid, and protein phases of the meat matrix [18, 19]. Therefore, findings obtained using fresh gooseberry pulp or paste cannot be directly extrapolated to freeze‐dried gooseberry.

Another limitation of existing studies is their focus on selected quality parameters, often without a comprehensive evaluation of physicochemical and redox‐related changes occurring during storage. Parameters such as oxidation–reduction potential (ORP), water activity, and total acidity, which characterize important aspects of the physicochemical environment of meat products, are still insufficiently studied in this context. Furthermore, systematic information on changes in these parameters throughout refrigerated storage remains limited, despite their technological relevance. In particular, antioxidant capacity determined in product extracts and the actual extent of lipid oxidation occurring within the meat matrix represent different but complementary aspects of oxidative stability. Simultaneous determination of antioxidant capacity, thiobarbituric acid reactive substances (TBARS), and ORP therefore enables the relationship between the antioxidant potential of the product and the actual oxidative response of the meat matrix during storage to be evaluated.

The present study addresses the limited knowledge concerning the functionality of freeze‐dried European gooseberry (*Ribes uva-crispa* L.) in complex meat matrices. The use of a concentrated, low‐moisture fruit material in a highly comminuted and heat‐processed pork pâté may influence both the physicochemical environment of the product and the course of oxidative processes during refrigerated storage. Simultaneous assessment of antioxidant capacity, lipid oxidation, ORP, acidity, and water activity provides a comprehensive characterization of storage‐related changes and enables the relationships between the antioxidant potential of the product, its redox conditions, and the progression of lipid oxidation to be evaluated. Such an integrated approach may contribute to a better understanding of the functional role of freeze‐dried *Ribes uva-crispa* in meat systems and its influence on the oxidative stability and physicochemical properties of pork pâté during storage.

The aim of the present study was to determine the effects of freeze‐dried European gooseberry (*Ribes uva-crispa* L.) incorporation on the physicochemical properties, oxidative stability, antioxidant potential, color, and texture of pork pâté during refrigerated storage. The physicochemical properties of the products were assessed by determining total acidity, water activity, and ORP, together with lipid oxidation, instrumental color, and texture. In addition, the antioxidant potential of aqueous extracts obtained from the products was determined using ABTS, DPPH, and reducing power (RP) assays. All analyses were conducted immediately after product preparation (Day 1) and after 7, 14, 21, and 28 days of refrigerated storage at 4°C. It was hypothesized that the incorporation of freeze‐dried European gooseberry would modify the physicochemical and redox characteristics of pork pâté and that changes in antioxidant potential could be associated with changes in lipid oxidation during refrigerated storage.

## 2. Materials and Methods

### 2.1. Raw Materials and Preparation of Experimental Pork Pâtés

Freeze‐dried European gooseberries (*Ribes uva-crispa* L.; country of origin: Poland) were purchased as a commercially available freeze‐dried product from a local retail store. The freeze‐dried fruits were ground in a colloid mill to obtain a fine powder, which was used as a functional additive in the experimental variants (Figure 1).

**Figure 1:**
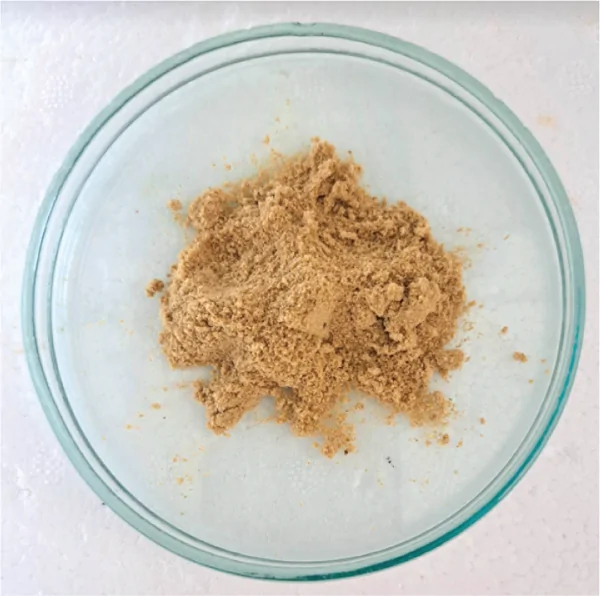
Freeze‐dried (powdered) *Ribes uva-crispa* gooseberries used in the experiment.

The raw meat was purchased from a local retail store in Lublin, Poland. Additional ingredients used for the pâté (spices, eggs, wheat bread, and raw onion) were purchased locally. The raw meat used for the pâté consisted of pork shoulder (60%), pork jowl (20%), and pork liver (20%). The raw meat was boiled in water at 100°C for 30 min. The resulting broth was collected and used in subsequent production steps. After cooling, the cooked meat was ground in a laboratory meat grinder equipped with a 3 mm diameter plate. The resulting meat batter was mixed with additional ingredients (egg, spices, onion, wheat breadcrumbs, and broth) according to the recipe presented in Table 1. The prepared meat batter was divided into four portions. Freeze‐dried gooseberry powder was then added, resulting in three experimental variants containing 0.5% (A0.5), 1% (A1), and 2% (A2) of the additive relative to the total mass of the meat batter. These addition levels corresponded to 5, 10, and 20 g of freeze‐dried gooseberry powder per 1000 g of meat batter, respectively. The control sample (AC) did not contain gooseberry powder. Aluminum molds (550 mL capacity) were filled with the prepared meat batter. The products were baked in the in a combi‐steam oven at 180°C for 50 min. After baking, the meat pâtés were cooled to approximately 18°C, vacuum‐packed, and stored under refrigerated conditions at 4°C until further analysis. Analyses were performed immediately after production (day 1) and after 7, 14, 21, and 28 days of storage of refrigerated storage.

**Table 1:** The composition of the prepared research variants.

Ingredients	AC	A0.5	A1	A2
Pork meat^*^	500 g	500 g	500 g	500 g
Broth	155 mL	155 mL	155 mL	155 mL
Egg	1	1	1	1
Salt	4.5 g	4.5 g	4.5 g	4.5 g
Pepper	0.5 g	0.5 g	0.5 g	0.5 g
Onion	60 g	60 g	60 g	60 g
Wheat roll	40 g	40 g	40 g	40 g
Freeze‐dried gooseberry^**^	0 g	5 g	10 g	20 g

^*^Pork meat consisted of pork shoulder, pork jowl, and pork liver in a ratio of 6:2:2.

^**^Amount per 1000 g of meat batter (g).

### 2.2. Evaluation of the Antioxidant Properties of Aqueous Gooseberry Extracts

#### 2.2.1. Preparation of Aqueous Extracts

Freeze‐dried gooseberry fruit powder was used to prepare aqueous extracts. The lyophilized material was mixed with distilled water (90°C) at a solid‐to‐solvent ratio of 1:10 (w/v) and extracted for 60 min with continuous shaking. After extraction, the mixture was centrifuged (30,000 rpm for 20 min) and the supernatant was filtered through filter paper. The determination of antioxidant activity was performed immediately after extract preparation.

#### 2.2.2. Total Reducing Capacity (TRC) by the Folin–Ciocalteu Assay

TRC was determined using the Folin–Ciocalteu method according to Singleton and Rossi [20]. Briefly, 100 *μ*L of extract was mixed with 100 *μ*L of distilled water and 400 *μ*L of Folin–Ciocalteu reagent (diluted 1:5 with distilled water). After 3 min, 2500 *μ*L of sodium carbonate solution (10% Na2CO₃) was added. The mixture was vortexed and incubated for 30 min at room temperature. A calibration curve was prepared using gallic acid standard solutions under identical conditions. Absorbance was measured at 725 nm using a spectrophotometer. Total phenolic content was calculated from the gallic acid calibration curve and expressed as *μ*g gallic acid equivalents per mL (*μ*g GAE/mL).

#### 2.2.3. RP Assay

The RP of the extracts was determined according to the method described by Oyaizu [21], based on the reduction of ferric ions (Fe^3+^) to ferrous ions (Fe^2+^). Briefly, 2 mL of the extract was mixed with 2 mL of sodium phosphate buffer (0.2 M, pH 6.6) and 2 mL of potassium ferricyanide solution (1%, K₃[Fe(CN)6]). The mixture was incubated in a water bath at 50°C for 20 min. Subsequently, 2 mL of trichloroacetic acid (10%) was added and the mixture was left to stand for 10 min. Then, 2 mL of the reaction mixture was transferred to a new tube and mixed with 2 mL of distilled water and 0.4 mL of ferric chloride solution (0.1% FeCl₃). After 10 min, absorbance was measured at 700 nm using a Nicolet Evolution 300 spectrophotometer (Thermo Electron Corp.). Higher absorbance values indicate greater RP of the extract.

#### 2.2.4. ABTS Radical Scavenging Activity

The antioxidant activity was evaluated using the ABTS radical cation (ABTS˙^+^) assay according to Re et al. [22]. The ABTS˙^+^ radical was generated by reacting 2,2 ^′^‐azino‐bis(3‐ethylbenzothiazoline‐6‐sulfonic acid) with potassium persulfate and incubating the mixture in the dark for 16 h. The ABTS solution was diluted with sodium phosphate buffer (0.02 M, pH 6.6) to obtain an absorbance of 0.70 ± 0.02 at 734 nm. The antioxidant activity was determined spectrophotometrically after 6 min of incubation of the extract with the ABTS solution at 734 nm. Radical scavenging activity was calculated according to the following equation:

Antioxidant activity%=1−As/Ac×100

where *As* – absorbance of the sample; *Ac* – absorbance of the control (ABTS solution).

#### 2.2.5. DPPH Radical Scavenging Activity

The free radical scavenging activity was determined using the DPPH assay according to Wu et al. [23] with some modifications. The method is based on the reduction of the purple methanolic solution of 2,2‐diphenyl‐1‐picrylhydrazyl (DPPH) by antioxidants present in the sample. Briefly, 1 mL of 0.2 mM DPPH solution in methanol was mixed with 200 *μ*L of extract and 800 *μ*L of distilled water. A mixture containing 1 mL of methanol and 1 mL of DPPH solution served as the control, while methanol alone was used as the blank. After 6 min of incubation in the dark, absorbance was measured at 517 nm. Radical scavenging activity was calculated using the following equation:

Antiradical  activity%=1−As/Ac×100

where *As* – absorbance of the sample; *Ac* – absorbance of the control (DPPH solution).

### 2.3. Evaluation of Physicochemical Properties of Pork Pâtés

#### 2.3.1. pH Measurement

The pH value was determined. Briefly, 10 g of sample was homogenized for 1 min with 100 mL of cold distilled water. The homogenate was filtered through filter paper, and pH was measured in the filtrate using a CPC‐501 digital pH/conductometer (Elmetron, Poland) equipped with a combined ERH‐111 electrode. Measurements were recorded with an accuracy of 0.01 after stabilization of the electrode signal. The pH meter was calibrated prior to analysis.

#### 2.3.2. ORP

ORP was determined using a platinum combined electrode (ERPt‐13) connected to a CPC‐501 pH/conductometer (Elmetron, Poland). Sample preparation was identical to that described for pH measurement. Results were expressed in millivolts (mV).

#### 2.3.3. Water Activity

Water activity (a_w_) was measured using a LabMaster‐aw instrument (Novasina AG, Lachen, Switzerland) equipped with a temperature‐controlled measurement chamber (20^°^C ± 1^°^C). Approximately 5 g of ground sample was placed in a measurement cup and inserted into the instrument chamber. The a_w_ value was read directly from the display after stabilization. Prior to analysis, the instrument was calibrated using Novasina humidity standards.

#### 2.3.4. Determination of TBARS

Lipid oxidation was evaluated by determining TBARS according to the method described by Pikul et al. [24]. Briefly, 3 g of ground sample was mixed with 200 *μ*L of alcoholic BHT solution (0.01%) and 12 mL of cold perchloric acid (4%, 4°C). The mixture was homogenized for 120 s using an Ultra‐Turrax homogenizer (T25 Basic, IKA, Germany). Samples were centrifuged at 6700 rpm for 8 min at 4°C and the supernatant was filtered through filter paper. Then, 650 *μ*L of filtrate was mixed with 650 *μ*L of TBA reagent (0.02 M) in 2 mL microtubes. Control samples containing 650 *μ*L of perchloric acid and 650 *μ*L of TBA reagent were prepared simultaneously. The mixtures were heated in a boiling water bath for 20 min, cooled to room temperature, and absorbance (Abs) was measured at 532 nm against the control sample. TBARS values were calculated according to the following equation:

TBARSmg MDA/kg product=Abs×5.5

### 2.4. Instrumental Evaluation of Product Quality

#### 2.4.1. Color Measurement (CIE *Lab*)

Instrumental color was measured using the CIE *Lab* color space, where *L*  ^∗^ represents lightness, *a*  ^∗^ indicates the red–green axis, and *b*  ^∗^ represents the yellow–blue axis [25].

Cube‐shaped samples (30 × 30 × 30 mm) were tightly wrapped in food‐grade film before measurement. Reflectance measurements were performed using a spherical spectrophotometer (X‐Rite) with a measurement aperture of 13 mm.

Illuminant D65 and a 10° standard observer were applied. The instrument was calibrated using a white reference standard before measurements. Each sample was measured five times and results were expressed as mean values.

#### 2.4.2. Texture Profile Analysis (TPA)

TPA was performed using a TA.XT Plus texture analyzer. Cylindrical samples (2 cm height) were obtained directly from the product using a cork borer. A double compression test was performed to 50% deformation at a probe speed of 5 mm/s with a 5‐s interval between compressions. Texture parameters were calculated automatically using the software integrated with the instrument. The following parameters were analyzed: hardness [N], springiness [mm], gumminess [N], chewiness [mJ], and cohesiveness [—].

### 2.5. Antioxidant Potential of Model Pork Pâtés Enriched With Freeze‐Dried Gooseberry

The antioxidant potential of the obtained products was evaluated using the ABTS, DPPH, and RP assays described in Section 2.2.

Prior to analysis, aqueous extracts were prepared by homogenizing pork pâté samples with distilled water at a ratio of 1:10 (w/v) using an Ultra‐Turrax homogenizer (T25 Basic, IKA, Germany) at 10,500 rpm for 2 min. The homogenate was centrifuged at 30,000 rpm for 10 min at 4°C. The obtained supernatant was filtered through filter paper and stored under refrigerated conditions until further analyses.

### 2.6. Data Analysis

Three independent production batches were prepared for each experimental formulation (AC, A0.5, A1, and A2), and each independently produced batch was considered an experimental unit (*n* = 3). At each storage time, three subsamples were collected from different locations within each production batch and analyzed for the respective parameters. The measurements obtained from these subsamples were averaged to provide a single value for each independent production batch. Results are presented as mean values ± standard deviation. The normality of data distribution was assessed using the Shapiro–Wilk test, while the homogeneity of variances was evaluated using Bartlett’s test. The main effects of freeze‐dried gooseberry addition (0%, 0.5%, 1%, and 2%) and storage time (1, 7, 14, 21, and 28 days), as well as their interaction (gooseberry addition × storage time), on the physicochemical, antioxidant, color, and texture parameters of pork pâtés were evaluated using two‐way analysis of variance (ANOVA). Significant differences between mean values were determined using Tukey’s post hoc test at a significance level of *p* < 0.05. Pearson’s correlation analysis was additionally performed to assess the relationships between ORP, TBARS, ABTS, and DPPH values. Correlations were considered statistically significant at *p* < 0.05. Statistical calculations were performed using Statistica software (version 13; StatSoft Inc., Tulsa, OK, United States).

## 3. Results

### 3.1. Assessment of the Antioxidant Potential of the Aqueous Gooseberry Extract

Prior to their application in pork pâté production, gooseberry fruits were evaluated for their antioxidant activity. The antioxidant potential of the extract was assessed using several complementary analytical methods that reflect different mechanisms of antioxidant action, including radical scavenging activity and reducing capacity. Using the Folin–Ciocalteu method, the TRC of the extract (often interpreted as an estimate of total phenolic content) was determined to be 38.80 *μ*g GAE per mL of extract (Table 2).

**Table 2:** Results of the evaluation of the antioxidant potential of the Aqueous Gooseberry extract.

Parameter	Value
Total reducing capacity [*μ*g GA/mL]	38.80 ± 3.10
Reducing power [A700]	1.84 ± 0.70
ABTS [%]	68.92 ± 1.25
DPPH [%]	35.01 ± 0.90

The analysis of antioxidant activity revealed that the tested extract exhibited higher radical scavenging activity against the ABTS radical compared with the DPPH radical, with values being almost twice as high in the ABTS assay. This difference may result from the distinct reaction mechanisms of the two assays, as the ABTS method allows the evaluation of both hydrophilic and lipophilic antioxidant compounds, whereas the DPPH assay is more selective toward lipophilic radical scavengers. Moreover, the extract demonstrated the ability to reduce Fe (III) ions, indicating the presence of compounds with reducing properties. Such activity suggests the presence of electron‐donating molecules, most likely phenolic compounds, which can terminate free‐radical chain reactions by donating hydrogen atoms or electrons, thereby converting reactive radicals into more stable forms.

### 3.2. Evaluation of Physicochemical Parameters of the Products

As shown in Table 3, the applied technological treatment, namely the addition of freeze‐dried gooseberry fruits, influenced the pH value of the pork pâtés. Overall, the incorporation of freeze‐dried gooseberry was associated with lower pH values compared with the control, although the magnitude and statistical significance of these differences varied with storage time. A concentration‐dependent decrease in pH was particularly evident on Days 1, 14, 21, and 28, whereas this relationship was not observed on Day 7. During refrigerated storage, pH values exhibited a time‐dependent pattern, with an overall increase relative to the initial measurements; however, the magnitude and direction of changes between consecutive storage intervals differed among the experimental formulations. Immediately after production (Day 1), an increase in the ORP was observed with increasing levels of freeze‐dried gooseberry. The highest ORP value was recorded in the sample containing 2% gooseberry (401.40 mV; A2), whereas the lowest value was observed in the control sample (372.57 mV; AC). However, these differences were not statistically significant at this stage of storage. During storage, a decrease in ORP was observed on Days 7 and 14, followed by an increase to values comparable to those observed immediately after production. The greatest fluctuations in ORP were observed in the A1 variant (1% addition). In this sample, ORP decreased from 394.27 mV (Day 1) to 309.53 mV (Day 7) and subsequently increased to 409.90 mV (Day 21), remaining relatively stable until the end of the storage period (Day 28). Additionally, on Days 7 and 14, all samples enriched with freeze‐dried gooseberry exhibited significantly higher ORP values than the control sample (*p* < 0.05).

**Table 3:** Total acidity (pH), ORP (oxidation–reduction potential), and water activity (a_w_) of model pork pâtés during storage (4°C).

Sample	Storage [days]				
	1	7	14	21	28
pH					
AC	5.73 ± 0.06 *^aA^*	5.95 ± 0.04 *^aB^*	6.53 ± 0.03 *^aC^*	6.30 ± 0.08 *^aD^*	6.36 ± 0.13 *^aCD^*
A0.5	5.50 ± 0.10 *^bA^*	5.59 ± 0.04 *^bA^*	6.26 ± 0.06 *^bB^*	6.01 ± 0.01 *^bC^*	6.18 ± 0.13 *^bBC^*
A1	5.30 ± 0.00 *^cA^*	5.93 ± 0.01 *^acB^*	5.94 ± 0.05 *^cB^*	5.76 ± 0.01 *^cC^*	5.84 ± 0.11 *^bBC^*
A2	4.90 ± 0.00 *^dA^*	5.87 ± 0.02 *^acB^*	5.56 ± 0.06 *^dC^*	5.27 ± 0.06 *^dD^*	5.36 ± 0.15 *^cCD^*
ORP					
AC	372.57 ± 28.12 *^aA^*	295.00 ± 5.15 *^aB^*	331.70 ± 1.47 *^aB^*	402.83 ± 1.76 *^aA^*	414.57 ± 17.17 *^aCA^*
A0.5	396.67 ± 0.75 *^aA^*	304.23 ± 1.52 *^bAB^*	335.37 ± 0.40 *^bAB^*	438.30 ± 58.46 *^aA^*	446.30 ± 40.65 *^aA^*
A1	394.27 ± 3.31 *^aA^*	309.53 ± 1.10 *^bcB^*	346.53 ± 1.66 *^cC^*	409.90 ± 2.09 *^aD^*	410.53 ± 1.82 *^aD^*
A2	401.40 ± 0.82 *^aA^*	313.43 ± 1.86 *^bB^*	352.50 ± 0.70 *^cC^*	402.57 ± 18.06 *^aA^*	411.70 ± 10.84 *^aA^*
a_w_					
AC	0.978 ± 0.001 *^aA^*	0.968 ± 0.002 *^aA^*	0.975 ± 0.001 *^aA^*	0.957 ± 0.017 *^aA^*	0.976 ± 0.002 *^aA^*
A0.5	0.976 ± 0.001 *^acA^*	0.959 ± 0.016 *^aA^*	0.976 ± 0.001 *^aA^*	0.965 ± 0.001 *^aA^*	0.968 ± 0.002 *^aA^*
A1	0.978 ± 0.001 *^abA^*	0.966 ± 0.001 *^aA^*	0.973 ± 0.001 *^aA^*	0.958 ± 0.016 *^aA^*	0.970 ± 0.014 *^aA^*
A2	0.974 ± 0.001 *^acA^*	0.967 ± 0.001 *^aA^*	0.972 ± 0.004 *^aA^*	0.964 ± 0.001 *^aA^*	0.978 ± 0.017 *^aA^*

*Note:* Mean values marked with different lowercase letters differ significantly (*p* < 0.05) at the same time point. Mean values marked with different uppercase letters differ significantly (*p* < 0.05) within the same sample. Results are expressed as mean ± standard deviation.

Abbreviations: AC, control sample; A0.5, 0.5% addition of freeze‐dried gooseberry; A1, 1% addition of freeze‐dried gooseberry; A2, 2% addition of freeze‐dried gooseberry.

Regarding water activity, no statistically significant differences were observed between the analyzed variants (*p* > 0.05). Moreover, storage time did not significantly affect this parameter. The only exception was observed on Day 1, when a statistically significant decrease in a_w_ was recorded in samples A0.5 and A2 compared with the A1 variant (*p* < 0.05). Nevertheless, the plant additive did not significantly affect water activity overall, as none of the enriched samples differed significantly from the control sample.

### 3.3. Instrumental Evaluation of Product Quality

#### 3.3.1. Evaluation of Color Parameters

Table 4 presents the color measurement results of the model pork pâtés expressed in the CIE*Lab* system.

**Table 4:** Color parameters of model pork pates during storage (4°C).

Sample	Storage [days]				
	1	7	14	21	28
Lightness (*L* ^∗^)					
AC	71.03 ± 1.76 *^aA^*	65.02 ± 3.33 *^aA^*	67.73 ± 0.92 *^aA^*	42.64 ± 5.93 *^aB^*	41.27 ± 7.19 *^aB^*
A0.5	71.03 ± 1.76 *^aA^*	65.67 ± 3.01 *^aA^*	66.39 ± 0.84 *^aA^*	42.98 ± 8.18 *^bB^*	43.21 ± 7.19 *^aB^*
A1	68.13 ± 1.05 *^bA^*	67.88 ± 3.65 *^aA^*	66.18 ± 1.42 *^aA^*	42.69 ± 6.83 *^bB^*	42.50 ± 7.63 *^aB^*
A2	65.41 ± 2.83 *^bA^*	66.04 ± 1.66 *^aA^*	67.26 ± 0.65 *^aA^*	43.72 ± 6.91 *^bB^*	43.09 ± 3.51 *^aB^*
Redness (*a* ^∗^)					
AC	3.17 ± 0.72 *^aA^*	4.45 ± 1.18 *^aA^*	5.92 ± 0.67 *^aA^*	5.94 ± 5.35 *^aA^*	6.93 ± 2.81 *^aA^*
A0.5	3.16 ± 0.72 *^aA^*	3.47 ± 1.10 *^aA^*	4.44 ± 0.49 *^bA^*	5.94 ± 5.35 *^aAB^*	6.43 ± 1.85 *^aAB^*
A1	4.51 ± 1.50 *^aA^*	4.81 ± 0.38 *^bA^*	4.11 ± 0.32 *^bA^*	7.05 ± 2.86 *^aAB^*	6.80 ± 2.45 *^aAB^*
A2	4.83 ± 1.07 *^aA^*	4.41 ± 0.90 *^aA^*	4.89 ± 0.86 *^bA^*	8.27 ± 2.14 *^aB^*	8.64 ± 2.01 *^aB^*
Yellowness (*b* ^∗^)					
AC	14.87 ± 4.16 *^aA^*	16.79 ± 1.84 *^aA^*	19.98 ± 0.66 *^aAB^*	9.22 ± 1.50 *^aB^*	9.28 ± 1.44 *^aB^*
A0.5	14.98 ± 4.03 *^aA^*	15.41 ± 2.49 *^aA^*	18.98 ± 1.12 *^aAB^*	9.48 ± 1.55 *^aB^*	9.32 ± 1.24 *^aB^*
A1	18.59 ± 0.68 *^aA^*	15.63 ± 1.76 *^aB^*	19.05 ± 0.88 *^aA^*	7.05 ± 2.86 *^aC^*	8.88 ± 2.01 *^aC^*
A2	19.88 ± 1.07 *^aA^*	16.76 ± 1.30 *^aB^*	19.20 ± 1.00 *^aA^*	9.15 ± 1.52 *^aC^*	9.05 ± 2.21 *^aC^*

*Note:* Mean values marked with different lowercase letters differ significantly (*p* < 0.05) at the same time point. Mean values marked with different uppercase letters differ significantly (*p* < 0.05) within the same sample. Results are expressed as mean ± standard deviation.

Abbreviations: AC, control sample; A0.5, 0.5% addition of freeze‐dried gooseberry; A1, 1% addition of freeze‐dried gooseberry; A2, 2% addition of freeze‐dried gooseberry.

The first parameter describing product color was lightness (*L*  ^∗^). Immediately after production (Day 1), a significant decrease (*p* < 0.05) in lightness was observed in the samples containing higher levels of freeze‐dried gooseberry (A1 and A2) compared with the control sample (71.03 in AC vs. 68.13 in A1 and 65.41 in A2).

On Day 21 of refrigerated storage, differences in *L*  ^∗^ values were observed between gooseberry‐enriched samples (A0.5, A1, A2) and the control sample (42.64 in AC compared with 42.98 in A0.5, 42.69 in A1, and 43.72 in A2). However, within the gooseberry‐enriched variants, no statistically significant differences were found between different levels of the additive (*p* > 0.05). A clear effect of storage time on lightness was also observed. A noticeable decrease in *L*  ^∗^ values was observed in all samples from Day 21 onward (e.g., from 71.03 to 41.27 in AC and from 65.41 to 43.09 in A2 between Day 1 and Day 28).

The second color parameter analyzed was *a*  ^∗^, representing the contribution of red color to the overall color tone (higher values indicate a more intense red color). As shown in Table 4, the effect of freeze‐dried gooseberry addition on the parameter was generally limited and depended on storage time. On Day 1, redness values ranged from 3.16 to 3.17 in AC and A0.5 to 4.51 in A1 and 4.83 in A2. Only on Day 14 was a significant decrease (*p* < 0.05) in redness observed in the gooseberry‐enriched samples (4.44 in A0.5, 4.11 in A1, and 4.89 in A2) compared with the control (5.92); however, this tendency was not maintained during the subsequent storage periods. At the end of storage (Day 28), the highest a value was recorded in A2 (8.64), followed by AC (6.93), A1 (6.80), and A0.5 (6.43).

A similar pattern was observed for the *b*  ^∗^ parameter, which reflects the presence of yellow color tones. The addition of gooseberry did not significantly influence *b*  ^∗^ values during the 28‐day storage period. On Day 1, *b*  ^∗^ values ranged from 14.87 in AC to 19.88 in A2. However, a considerable decrease in *b*  ^∗^ values was observed after Day 14 in all analyzed variants (e.g., from 19.98 to 9.28 in AC and from 19.20 to 9.05 in A2 between Day 14 and Day 28).

#### 3.3.2. Evaluation of Texture Parameters

Based on the data presented in Table 5, the addition of freeze‐dried gooseberry fruit at levels of 0.5%, 1%, and 2% did not significantly affect the hardness of pork pâtés.

**Table 5:** Texture parameters: hardness [N], springiness [mm], gumminess [N], chewiness [mJ], and cohesiveness [‐] of model pork pâtés during storage (4°C).

Sample	Storage [days]				
	1	7	14	21	28
Hardness [N]					
AC	9.34 ± 1.29 *^aA^*	17.99 ± 1.46 *^aB^*	30.94 ± 2.81 *^aC^*	26.78 ± 5.56 *^aCD^*	24.95 ± 1.63 *^aD^*
A0.5	10.10 ± 1.79 *^aA^*	18.96 ± 0.46 *^aB^*	34.35 ± 2.81 *^aC^*	25.17 ± 0.56 *^aD^*	29.53 ± 1.24 *^aD^*
A1	11.68 ± 1.35 *^aA^*	19.77 ± 1.19 *^aB^*	31.52 ± 0.19 *^aC^*	25.80 ± 4.55 *^aD^*	23.88 ± 3.36 *^aBD^*
A2	9.70 ± 1.22 *^aA^*	17.44 ± 2.47 *^aB^*	30.81 ± 1.86 *^aC^*	29.75 ± 3.45 *^aC^*	27.20 ± 2.38 *^aC^*
Springiness [mm]					
AC	0.630 ± 0.07 *^aA^*	0.364 ± 0.04 *^aB^*	0.552 ± 0.03 *^aA^*	0.342 ± 0.03 *^aB^*	0.339 ± 0.01 *^aB^*
A0.5	0.656 ± 0.01 *^aA^*	0.451 ± 0.05 *^bB^*	0.551 ± 0.03 *^aA^*	0.345 ± 0.06 *^aC^*	0.346 ± 0.05 *^aC^*
A1	0.521 ± 0.09 *^aAB^*	0.395 ± 0.05 *^cA^*	0.593 ± 0.07 *^bB^*	0.401 ± 0.03 *^bA^*	0.406 ± 0.01 *^bA^*
A2	0.642 ± 0.02 *^aA^*	0.352 ± 0.04 *^aB^*	0.519 ± 0.03 *^aC^*	0.345 ± 0.02 *^aB^*	0.355 ± 0.03 *^aB^*
Gumminess [N]					
AC	2.45 ± 0.34 *^aA^*	4.23 ± 0.60 *^aA^*	8.19 ± 0.51 *^aB^*	5.79 ± 0.60 *^aA^*	6.19 ± 0.51 *^aA^*
A0.5	3.37 ± 0.55 *^aA^*	3.93 ± 0.38 *^aA^*	6.82 ± 0.51 *^aB^*	6.77 ± 0.21 *^bC^*	6.78 ± 0.22 *^aC^*
A1	4.99 ± 0.66 *^bA^*	4.09 ± 0.61 *^aA^*	5.99 ± 0.79 *^aB^*	5.41 ± 0.32 *^aA^*	5.73 ± 0.44 *^aA^*
A2	2.93 ± 0.49 *^aA^*	3.62 ± 0.31 *^aA^*	5.47 ± 0.78 *^aB^*	6.34 ± 0.04 *^abC^*	6.33 ± 0.21 *^aC^*
Chewiness [mJ]					
AC	175.82 ± 26.18 *^aA^*	422.89 ± 60.17 *^aB^*	813.34 ± 70.03 *^aC^*	206.67 ± 38.91 *^aA^*	210.61 ± 36.06 *^aA^*
A0.5	220.71 ± 32.07 *^abA^*	393.20 ± 37.58 *^aB^*	792.82 ± 33.26 *^aC^*	231.50 ± 24.98 *^aA^*	235.94 ± 31.31 *^aA^*
A1	264.12 ± 35.67 *^bA^*	442.60 ± 3.77 *^aB^*	679.28 ± 15.39 *^aC^*	232.51 ± 7.26 *^aA^*	233.94 ± 4.73 *^aA^*
A2	188.74 ± 37.36 *^abA^*	362.50 ± 30.90 *^aB^*	487.80 ± 17.64 *^bC^*	250.67 ± 13.58 *^aA^*	227.61 ± 15.57 *^aA^*
Cohesiveness [—]					
AC	0.271 ± 0.02 *^aA^*	0.234 ± 0.02 *^aA^*	0.420 ± 0.02 *^aB^*	0.253 ± 0.04 *^aA^*	0.253 ± 0.03 *^aA^*
A0.5	0.335 ± 0.02 *^aA^*	0.207 ± 0.02 *^aB^*	0.386 ± 0.02 *^aC^*	0.261 ± 0.04 *^aB^*	0.269 ± 0.03 *^aAB^*
A1	0.444 ± 0.05 *^bA^*	0.208 ± 0.05 *^aB^*	0.471 ± 0.02 *^aA^*	0.243 ± 0.04 *^aB^*	0.252 ± 0.03 *^aB^*
A2	0.301 ± 0.01 *^aA^*	0.207 ± 0.01 *^aB^*	0.355 ± 0.02 *^aC^*	0.247 ± 0.04 *^aB^*	0.244 ± 0.03 *^aB^*

*Note:* Mean values marked with different lowercase letters differ significantly (*p* < 0.05) at the same time point. Mean values marked with different uppercase letters differ significantly (*p* < 0.05) within the same sample. Results are expressed as mean ± standard deviation.

Abbreviations: AC, control sample; A0.5, 0.5% addition of freeze‐dried gooseberry; A1, 1% addition of freeze‐dried gooseberry; A2, 2% addition of freeze‐dried gooseberry.

Based on the data presented in Table 5, the addition of freeze‐dried gooseberry fruits at levels of 0.5%, 1%, and 2% did not significantly affect the hardness of pork pâtés. On Day 1, hardness values ranged from 9.34 N in AC to 11.68 N in A1.

However, storage time significantly influenced this parameter. Hardness increased gradually during storage, reaching the highest values on Day 14 (30.94 N in AC, 34.35 N in A0.5, 31.52 N in A1 and 30.81 N in A2), followed by a decrease in subsequent storage periods (e.g., to 24.95 N in AC and 23.88 N in A1 on Day 28). The only exception was the A2 variant, in which hardness remained relatively stable between 30.81 N (Day 14) and 27.20 N (Day 28).

The springiness of the products was also evaluated. Immediately after production (Day 1), the addition of gooseberry did not significantly affect springiness (*p* > 0.05), with values of (0.630 in AC, 0.656 in A0.5, 0.521 in A1 and 0.642 in A2). On Day 7, a substantial decrease in springiness was observed in all samples (0.364 in AC, 0.451 in A0.5, 0.395 in A1 and 0.352 in A2); however, this decrease was less pronounced in the A0.5 and A1 variants, which differed significantly from the control sample (*p* < 0.05). After 14 days of storage, springiness increased markedly (0.552 in AC, 0.551 in A0.5, 0.593 in A1 and 0.519 in A2), followed by a subsequent decrease in later storage periods (*p* < 0.05). No significant difference was observed between Days 21 and 28 (*p* > 0.05). At the end of the storage period, the A1 variant (1% gooseberry) exhibited significantly higher springiness (0.406) compared with the other samples (0.339 in AC, 0.346 in A0.5 and 0.355 in A2).

The effect of freeze‐dried gooseberry addition on cohesiveness was generally limited and depended on storage time. On Day 1, the A1 variant exhibited the highest cohesiveness value (0.444), which was significantly higher than those recorded for AC (0.271), A0.5 (0.335), and A2 (0.301) (*p* < 0.05). At subsequent storage times, no significant differences in cohesiveness were observed among the formulations (*p* > 0.05). However, a significant decrease in cohesiveness between Day 1 and Day 28 was observed in A1 (from 0.444 to 0.252) and A2 (from 0.301 to 0.244) (*p* < 0.05).

The effect of freeze‐dried gooseberry addition on gumminess depended on storage time. On Day 1, the A1 variant exhibited significantly higher gumminess (4.99 N) than AC (2.45 N), A0.5 (3.37 N), and A2 (2.93 N) (*p* < 0.05). No significant differences among the formulations were observed on Days 7 and 14 (*p* > 0.05). On Day 21, A0.5 exhibited significantly higher gumminess (6.77 N) than AC (5.79 N) and A1 (5.41 N), whereas A2 (6.34 N) did not differ significantly from either group. On Day 28, no significant differences among the formulations were observed (*p* > 0.05). During storage, gumminess generally increased from the initial values, although the pattern of changes differed among formulations. The highest values were recorded on Day 14 for AC (8.19 N) and on Days 21–28 for A0.5 and A2.

The last analyzed texture parameter was chewiness. The effect of freeze‐dried gooseberry addition on chewiness was dependent on storage time. On Day 1, the A1 variant exhibited the highest chewiness value (264.12 mJ) and was significantly higher than the control sample (175.82 mJ; *p* < 0.05), whereas A0.5 (220.71 mJ) and A2 (188.74 mJ) did not differ significantly from either AC or A1. No significant differences among the formulations were observed on Day 7. On Day 14, the A2 variant exhibited significantly lower chewiness (487.80 mJ) than AC (813.34 mJ), A0.5 (792.82 mJ), and A1 (679.28 mJ) (*p* < 0.05). No significant differences among the formulations were observed on Days 21 and 28 (*p* > 0.05).

### 3.4. Evaluation of the Antioxidant Status of Pork Pâtés

#### 3.4.1. Determination of TBARS

The content of TBARS, which are secondary products of lipid oxidation formed mainly during the decomposition of polyunsaturated fatty acid hydroperoxides, is presented in Table 6. The TBARS assay quantifies malondialdehyde (MDA) and other thiobarbituric acid reactive compounds, which are widely used indicators of the intensity of oxidative degradation of lipids in meat products.

**Table 6:** TBARS level of model pork pates during storage (4°C).

Sample	Storage [days]				
	1	7	14	21	28
AC	1.70 ± 0.25 *^aA^*	1.80 ± 0.43 *^aA^*	2.30 ± 0.57 *^aA^*	1.80 ± 0.29 *^aA^*	2.00 ± 0.61 *^aAB^*
A0.5	1.60 ± 0.39 *^aA^*	2.10 ± 0.19 *^aB^*	2.30 ± 0.20 *^aB^*	2.00 ± 0.22 *^aAB^*	2.00 ± 0.55 *^aAB^*
A1	1.30 ± 0.33 *^aA^*	1.40 ± 0.37 *^aB^*	2.30 ± 0.29 *^aB^*	1.90 ± 0.70 *^aB^*	2.00 ± 0.25 *^aB^*
A2	1.90 ± 0.71 *^aA^*	1.50 ± 0.33 *^aB^*	2.00 ± 0.35 *^aA^*	1.30 ± 0.22 *^aB^*	1.40 ± 0.35 *^aB^*

*Note:* Mean values marked with different lowercase letters differ significantly (*p* < 0.05) at the same time point. Mean values marked with different uppercase letters differ significantly (*p* < 0.05) within the same sample. Results are expressed as mean ± standard deviation.

Abbreviations: AC, control sample; A0.5, 0.5% addition of freeze‐dried gooseberry; A1, 1% addition of freeze‐dried gooseberry; A2, 2% addition of freeze‐dried gooseberry.

The content of TBARS in the analyzed pork pâtés during refrigerated storage is presented in Table 6. On the first day of storage, TBARS values ranged from 1.30 mg MDA/kg in sample A1 to 1.90 mg MDA/kg in sample A2, while the control sample (AC) exhibited a value of 1.70 mg MDA/kg. After 7 days of storage, TBARS values varied between 1.40 mg MDA/kg (A1) and 2.10 mg MDA/kg (A0.5), whereas the control sample reached 1.80 mg MDA/kg. On Day 14, an increase in TBARS values was observed in all analyzed variants. At this time point, the highest TBARS value (2.30 mg MDA/kg) was recorded in samples AC, A0.5, and A1, while a lower value was observed in sample A2 (2.00 mg MDA/kg). In the subsequent storage period, a decrease in TBARS values was recorded. On Day 21, TBARS values ranged from 1.30 mg MDA/kg in sample A2 to 2.00 mg MDA/kg in sample A0.5, while the control sample showed a value of 1.80 mg MDA/kg. At the end of storage (Day 28), TBARS values ranged between 1.40 mg MDA/kg (A2) and 2.00 mg MDA/kg (AC, A0.5, and A1). No statistically significant differences in TBARS values were observed among the formulations at any of the individual storage times (*p* > 0.05), indicating that freeze‐dried gooseberry addition did not significantly affect the formation of secondary lipid oxidation products under the conditions of the present study. Significant changes in TBARS values during storage were observed within some formulations. In particular, TBARS values on Day 14 were significantly higher than those recorded on day 1 in the A0.5 and A1 variants (*p* < 0.05), whereas no significant difference between these time points was observed for AC or A2.

#### 3.4.2. Determination of the Antioxidant Activity of Pork Pâtés

The addition of freeze‐dried gooseberry significantly influenced the antiradical activity of pork pâtés against the ABTS˙^+^ radical cation (Table 7). On the first day of storage, ABTS radical scavenging activity in the control sample was 35.3%, whereas significantly higher values were recorded in A1 (51.3%) and A2 (52.3%) (*p* < 0.05). In contrast, the A0.5 variant (33.0%) did not differ significantly from the control sample (*p* > 0.05). After 7 days of storage, ABTS activity increased to 61.4% in the A2 sample, while lower values were recorded in the remaining variants (37.0% in AC, 33.0% in A0.5, and 48.0% in A1). The highest ABTS activity was observed on Day 14 when sample A2 reached 84.7%, whereas the control sample and A0.5 exhibited lower values of 34.0% and 24.5%, respectively. In the later stages of storage, ABTS activity decreased from the maximum observed on Day 14; however, the A2 variant continued to exhibit higher values than the control sample. On Day 21, ABTS activity reached 62.7% in A2 compared with 38.7% in the control sample, while on Day 28, the values were 60.7% and 35.3%, respectively. The analyzed samples also exhibited radical scavenging activity toward DPPH radicals. On Day 1, DPPH activity ranged from 25.1% in sample A2 to 35.5% in the control sample. After 7 days of storage, a decrease in DPPH values was observed in all variants, with values reaching 14.5% in AC, 15.6% in A0.5, 9.8% in A1, and 11.3% in A2. On day 14, an increase in DPPH scavenging activity was observed in samples containing gooseberry, reaching 33.0% in A0.5 and 32.5% in A1, whereas the control sample exhibited a lower value of 13.8%. In the subsequent storage period, DPPH values decreased again in most samples. At the end of storage (Day 28), the lowest value was recorded in sample A1 (9.0%), while sample A0.5 exhibited the highest value (20.3%). The RP of the analyzed samples expressed as absorbance at 700 nm (A700) did not differ significantly between variants. On Day 1, RP values ranged from 1.30 (A1) to 1.90 (A2), while the control sample exhibited a value of 1.70. During refrigerated storage, RP varied over time within the individual formulations, with values reaching approximately 2.0–2.3 on day 14. However, no statistically significant differences in RP were observed among the formulations at any of the individual storage times (*p* > 0.05).

**Table 7:** Antioxidant potential of model pork pates during storage (4°C).

Sample	Storage [days]				
	1	7	14	21	28
ABTS [%]					
AC	35.3 ± 3.21 *^aA^*	37.0 ± 1.0 *^aA^*	34.0 ± 8.54 *^aA^*	38.7 ± 1.53 *^aA^*	35.3 ± 5.13 *^aA^*
A0.5	33.0 ± 7.21 *^aA^*	33.0 ± 2.65 *^aA^*	24.5 ± 1.32 *^bA^*	39.3 ± 0.58 *^aB^*	36.3 ± 2.52 *^aA^*
A1	51.3 ± 1.15 *^bA^*	48.0 ± 2.0 *^bA^*	55.3 ± 2.31 *^cA^*	34.0 ± 1.73 *^bB^*	40.3 ± 5.86 *^aB^*
A2	52.3 ± 3.79 *^bA^*	61.4 ± 4.13 *^cB^*	84.7 ± 0.58 *^dC^*	62.7 ± 0.58 *^cB^*	60.7 ± 3.79 *^bB^*
DPPH [%]					
AC	35.5 ± 4.2 *^aA^*	14.5 ± 2.1 *^aB^*	13.8 ± 2.2 *^aB^*	10.7 ± 0.6 *^aB^*	9.8 ± 1.9 *^aB^*
A0.5	25.5 ± 1.0 *^bA^*	15.6 ± 1.9 *^aB^*	33.0 ± 4.4 *^bC^*	27.0 ± 4.1 *^bAC^*	20.3 ± 2.1 *^bAB^*
A1	31.5 ± 5.4 *^abA^*	9.8 ± 0.4 *^bB^*	32.5 ± 0.7 *^bA^*	20.6 ± 0.5 *^cC^*	9.0 ± 1.6 *^aAC^*
A2	25.1 ± 1.2 *^bA^*	11.3 ± 1.7 *^abB^*	26.5 ± 3.0 *^bA^*	23.2 ± 2.3 *^bcA^*	12.0 ± 1.6 *^aB^*
Reducing power [A700]					
AC	1.70 ± 0.25 *^aA^*	1.80 ± 0.43 *^aA^*	2.3 ± 0.57 *^aA^*	1.80 ± 0.29 *^aA^*	2.0 ± 0.61 *^aA^*
A0.5	1.60 ± 0.39 *^aA^*	2.1 ± 0.19 *^aA^*	2.3 ± 0.20 *^aA^*	2.0 ± 0.22 *^aA^*	2.0 ± 0.55 *^aA^*
A1	1.30 ± 0.33 *^aA^*	1.4 ± 0.37 *^aA^*	2.3 ± 0.29 *^aB^*	1.90 ± 0.70 *^aAB^*	2.0 ± 0.25 *^aAB^*
A2	1.90 ± 0.71 *^aA^*	1.4 ± 0.37 *^aA^*	2.0 ± 0.35 *^aB^*	1.30 ± 0.22 *^aA^*	1.40 ± 0.35 *^aA^*

*Note:* Mean values marked with different lowercase letters differ significantly (*p* < 0.05) at the same time point. Mean values marked with different uppercase letters differ significantly (*p* < 0.05) within the same sample. Results are expressed as mean ± standard deviation.

Abbreviations: AC, control sample; A0.5, 0.5% addition of freeze‐dried gooseberry; A1, 1% addition of freeze‐dried gooseberry; A2, 2% addition of freeze‐dried gooseberry.

## 4. Discussion

In recent years, increasing scientific interest has focused on the development of foods with enhanced health‐promoting properties. This trend is reflected in the growing number of studies focusing on reducing the risk of diet‐related diseases caused, among other things, by the consumption of low‐quality food, and on incorporating biologically active compounds into food products. Among these compounds, natural antioxidants are particularly desirable. Because of the high content of these compounds in plant materials, food producers are increasingly adding herbs, fruits, and vegetables to animal products, especially meat. A similar approach has been widely described in studies on the use of gooseberry‐derived ingredients, particularly Indian gooseberry (*Phyllanthus emblica* L.), as natural functional additives in meat products, where their ability to improve oxidative stability and storage quality has been demonstrated [13, 16]. Generally, plant‐derived ingredients are a rich source of phenolic compounds and other antioxidant molecules that can delay lipid and protein oxidation, thus improving the quality and shelf life of meat products and providing a natural alternative to synthetic food additives [26]. Recent studies have further demonstrated that medicinal and aromatic plant extracts, fruit‐derived ingredients, and essential oils may exhibit antioxidant and other functional effects in meat‐related systems, although their effectiveness depends strongly on botanical source, concentration, method of application, and food matrix [44–50].

Water activity is an important physicochemical characteristic of meat products and represents one of several factors influencing microbial growth and storage‐related quality changes. In this study, water activity ranged from 0.957 to 0.978 depending on the experimental variant and storage time. The addition of freeze‐dried gooseberries did not significantly affect this parameter. Similar observations were noted in studies of chicken nuggets enriched with gooseberry pulp and seed coat powder, where no significant changes in water activity were observed despite the presence of plant additives [13]. Studies of restructured poultry products with the addition of gooseberries, tomatoes, and red grapes also showed no significant effect of fruit additives on water activity [15, 17]. According to Fontana [27], water activity values below 0.980 may restrict the growth of some microorganisms associated with meat spoilage. However, water activity represents only one of several factors governing microbial growth in meat products, and the values obtained in the present study cannot be interpreted as evidence of microbiological stability in the absence of direct microbiological analyses.

Another important quality parameter of meat products is pH. The results obtained in this study confirmed that both gooseberry addition and storage time influenced the pH values of model pork pâtés. Overall, gooseberry‐enriched samples exhibited lower pH values than the control, although the magnitude and statistical significance of these differences varied with storage time. A concentration‐dependent decrease in pH was particularly evident on Days 1, 14, 21, and 28, whereas this relationship was less apparent on Day 7. A similar trend was observed by Goswami et al. [28], who noted a gradual decrease in pH in chicken nuggets with increasing dried gooseberry pulp content (0.5%–1.5%) [13]. The observed decrease in pH with increasing plant addition level can be attributed to the presence of organic acids naturally occurring in gooseberries. Similar observations were reported by Bellucci et al. [29], who demonstrated that the addition of freeze‐dried plant materials to meat products can lead to a decrease in pH. In that study, pH values gradually decreased with increasing dried gooseberry flesh content (0.5%–1.5%), whereas this relationship was not observed when powdered gooseberry peel was used, indicating that different anatomical parts of the fruit may exert different effects on the meat matrix. Similar results have also been described in studies on functional mutton products, in which plant additives contributed to the acidification of the meat matrix [16]. A similar pH reduction was also observed in studies on poultry meatballs with added gooseberry paste, indicating that this effect may also occur in other types of meat products [11]. These results support the view that the intrinsic acidity and organic acid composition of fruit‐derived ingredients may substantially influence the pH of meat matrices.

ORP, which reflects the overall balance between oxidizing and reducing components within the food matrix [30], showed a numerical tendency to increase with increasing gooseberry content immediately after production, although the differences among formulations were not statistically significant. Higher ORP values typically indicate a greater presence of oxidizing agents. However, it should be noted that meat products are naturally susceptible to oxidation processes. Thermal processing can disrupt muscle cell structures, inactivate endogenous antioxidant enzymes, and promote the release of iron from myoglobin molecules, thus creating a pro‐oxidant environment [6]. Similar complexity has been observed in studies involving gooseberry‐derived ingredients, where the presence of antioxidant compounds does not necessarily result in measurable inhibition of oxidative processes in the meat matrix [10]. In plant systems, phenolic compounds can exhibit both antioxidant and prooxidant properties, depending on environmental conditions and the presence of metal ions. For example, flavonoids and dihydroxycinnamic acids can generate free radicals in the presence of oxygen and transition metals, leading to the formation of phenoxy radicals capable of initiating lipid peroxidation [31, 32]. This phenomenon may partially explain the results obtained in the present study, in which gooseberry addition did not significantly reduce the formation of secondary lipid oxidation products. TBARS values did not differ significantly between experimental variants despite the presence of antioxidant compounds in the plant material. To further evaluate whether the observed changes in ORP were associated with lipid oxidation or antioxidant capacity, Pearson correlation analysis was performed between ORP and TBARS, ABTS, and DPPH values. No significant correlations were found between ORP and TBARS (r = −0.106, *p* = 0.657), ORP and ABTS (r = −0.044, *p* = 0.854), or ORP and DPPH (r = 0.111, *p* = 0.643). These results indicate that the fluctuations in ORP were not directly associated with either the formation of secondary lipid oxidation products or the radical‐scavenging activity determined in the product extracts. This lack of association may be explained by the complex nature of ORP in meat systems, as this parameter reflects the overall balance between numerous oxidizing and reducing components present in the matrix rather than lipid oxidation alone. In contrast, TBARS specifically reflects the accumulation of secondary lipid oxidation products, whereas ABTS and DPPH characterize the radical‐scavenging capacity of extractable antioxidant compounds. Consequently, changes in these parameters may occur independently during storage, which is consistent with the absence of significant correlations observed in the present study. However, some studies have shown a beneficial effect of gooseberry additives on lipid oxidation in meat systems. For example, Argade et al. [16] reported that mutton rolls enriched with Indian gooseberry extract exhibited significantly lower TBARS values compared to control samples. Similar antioxidant effects of plant additives in meat products were described by Wójciak et al. [33], Manessis [34], and Tocai et al. [35]. It is also worth noting that in studies on poultry nuggets and meatballs gooseberry pulp or paste effectively limited lipid oxidation, which suggests that form may play a key role in its functional activity [11, 13]. Comparable effects have been reported for other plant‐derived antioxidant systems. Yerlikaya and Şen Arslan [36] reported that ethanolic extracts of propolis, sage (*Salvia officinalis* L.), and lavender (*Lavandula angustifolia* Mill.) reduced lipid oxidation in chicken sausages compared with the control. The lack of a significant TBARS reduction in the present study, despite the measurable antioxidant capacity of the gooseberry‐containing extracts, therefore differs from the responses observed in several extract‐based systems. This discrepancy may partly reflect differences in the physical form and effective concentration of the antioxidant material. Ethanolic extracts, seed extracts, and fruit juices may provide more readily accessible antioxidant compounds than whole freeze‐dried fruit powder incorporated into a complex, heat‐processed meat matrix. Differences between studies may result from differences in the concentration and composition of bioactive compounds in plant materials, their physical form and method of application, as well as the composition and processing conditions of the meat matrix.

Plant additives may also influence the color and texture of meat products. Changes in color parameters may occur due to the presence of pigments, phenolic compounds, or dietary fiber in plant materials [2, 35]. In the present study, the addition of gooseberry influenced the lightness parameter (*L*  ^∗^) of the model product. Similar observations were reported by Wójciak et al. [33], who found that the incorporation of plant extracts such as green tea and red pepper resulted in significant changes in the lightness of meat products. Studies on sausages and restructured products have shown that the addition of gooseberries improved color stability during storage, which was attributed to the antioxidant activity of phenolic compounds. Argade et al. [15] found that the inclusion of gooseberries in functional lamb rolls had a positive effect on the texture and color profile of the product. Ribas et al. [10] demonstrated that Ceylon gooseberries as an addition to meat cutlets improved their sensory and instrumental quality. However, in the present study, gooseberry addition did not consistently affect redness (*a*  ^∗^) or yellowness (*b*  ^∗^) throughout storage. A transient decrease in redness was observed after 14 days of storage in samples enriched with gooseberries. Interestingly, this time point also corresponded to the highest TBARS values observed during storage, indicating a numerical increase in the accumulation of secondary lipid oxidation products at this stage of storage. Lipid oxidation is known to initiate the oxidation of heme pigments, which may lead to a reduction in red color intensity in meat products [37]. Nevertheless, because TBARS values did not differ significantly among formulations at individual storage times, the differences in color cannot be attributed exclusively to differences in lipid oxidation. Direct effects of plant pigments, acidity, and interactions of phenolic compounds with the meat matrix may also have contributed to the observed color changes.

Another important aspect of plant additives in meat products is their effect on texture. Instrumental texture analysis indicated that the effect of freeze‐dried gooseberry on the texture of pork pâté was generally limited but depended on both the texture parameter and storage time. Hardness was not significantly affected by gooseberry addition, whereas significant differences among formulations were observed at selected storage times for springiness, cohesiveness, gumminess, and chewiness. Storage time also markedly influenced several texture parameters. In general, the introduction of plant materials in the form of powders, freeze‐dried products, or fresh tissues can affect texture due to the presence of dietary fiber. Plant fibers are widely studied for their functional properties in meat products, including water retention, gel formation, viscosity, solubility, and structural stabilization, particularly in reduced‐fat preparations. Other studies have shown that the addition of fiber sources such as pea, bamboo, or sugarcane fiber can significantly increase the firmness of poultry products, while citrus and apple fibers have little or no effect [38]. However, studies on mutton products and meat patties with gooseberry addition noted changes in textural parameters such as hardness and cohesiveness, suggesting that this effect depends on the product type and the form of the addition [15, 16]. Such variability suggests that plant species, processing methods, and fiber composition strongly influence interactions with meat proteins and water retention capacity in meat systems [39, 40]. In this context, other authors emphasized that fruit‐ and vegetable‐derived dietary fibers can modify the technological properties of meat products through their water‐binding capacity and effects on texture and storage‐related characteristics [38–40]. Importantly, the magnitude and direction of these changes depend on the origin and physicochemical properties of the fiber source. This may explain why the texture response observed in the present study was relatively limited and parameter‐dependent rather than uniform across all texture attributes. These observations support the interpretation that plant‐derived ingredients may selectively modify individual textural properties rather than produce a general change across all texture attributes.

This study also assessed the antioxidant potential of gooseberry‐enriched pork pâtés. The ABTS assay revealed a pronounced increase in antiradical activity in gooseberry‐enriched samples, particularly at the highest inclusion level (2%), whereas the response observed in the DPPH assay was more variable and depended on both gooseberry concentration and storage time. This effect is consistent with the presence of phenolic compounds, flavonoids, and vitamin C in gooseberries, which contribute to their antioxidant capacity. Consistent observations have been reported for other plant‐derived ingredients used in meat systems. For example, Ramli et al. [41] demonstrated that passion fruit peel extract added to meat products exhibited significant antioxidant activity in ABTS, DPPH, and RP assays. Comparable findings were also obtained in studies on gooseberry meat products, where significant antioxidant activity was observed with increasing supplement levels [11–13, 18]. The variability between ABTS and DPPH responses observed in the present study is not unusual for plant‐derived antioxidant systems. Different antioxidant assays are based on different reaction mechanisms and analytical conditions; therefore, the antioxidant capacity measured in meat systems may vary depending on the assay used and the chemical characteristics of the antioxidant compounds [42, 43]. Accordingly, antioxidant capacity should be interpreted using complementary analytical approaches rather than on the basis of a single chemical assay [42]. These considerations may help explain the differences between the ABTS and DPPH responses observed in the present study. Importantly, the increased antiradical activity observed in the present study was not accompanied by a significant reduction in TBARS values between the experimental variants. This discrepancy may be explained by the fact that ABTS and DPPH assays characterize the radical‐scavenging capacity of extractable compounds under defined analytical conditions, whereas TBARS reflects the formation of secondary lipid oxidation products within the complex meat matrix. Therefore, an increase in extractable antioxidant capacity does not necessarily indicate that the active compounds are present at sufficient concentrations or are sufficiently available at the sites of lipid oxidation to inhibit this process effectively. In a meat matrix, phenolic compounds may interact with proteins and other macromolecules, which can alter their extractability, accessibility, and antioxidant effectiveness [44, 45]. Furthermore, thermal processing may modify or degrade some antioxidant compounds, while simultaneously promoting pro‐oxidative conditions through disruption of cellular structures, inactivation of endogenous antioxidant enzymes, and release of pro‐oxidant iron from heme proteins [6]. The levels of freeze‐dried gooseberry used in the present study increased the measurable antiradical capacity of aqueous extracts, but this effect was not accompanied by a statistically significant reduction in lipid oxidation in the pâté matrix. The effectiveness of plant‐derived antioxidants in meat systems depends on several factors, including the botanical source and composition of the antioxidant material, concentration of bioactive compounds, processing conditions, method of application, analytical approach, and interactions with the meat matrix [42, 43]. Similar variability in the antioxidant response of meat systems supplemented with plant‐derived ingredients has also been reported for antioxidant‐rich fruit and vegetable juices and selected plant extracts [36, 46]. Consequently, antioxidant effectiveness against lipid oxidation cannot be inferred solely from the antioxidant capacity measured in extracts. These results demonstrate the importance of distinguishing between antioxidant capacity measured by chemical radical‐scavenging assays and antioxidant effectiveness against lipid oxidation in the food matrix. Accordingly, the present results indicate that freeze‐dried European gooseberry increased the measurable antioxidant potential of pork pâté extracts; however, this effect was not accompanied by significant differences in TBARS values among the experimental formulations and therefore cannot be interpreted as evidence of improved lipid oxidative stability under the conditions of the present study.

## 5. Conclusions

The incorporation of 0.5%–2% freeze‐dried European gooseberry (*Ribes uva-crispa* L.) did not produce consistent adverse changes in the physicochemical and textural characteristics of pork pâté, although selected texture parameters differed among formulations at individual storage times. Increasing gooseberry addition was associated with a decrease in pH, which may be attributed to the presence of naturally occurring organic acids in the fruit. The instrumental color parameters were generally only slightly affected by gooseberry incorporation, although changes in lightness (*L* ^?^) and a transient decrease in redness (*a* ^?^) were observed during storage. Freeze‐dried gooseberry significantly increased the antioxidant potential of pork pâté extracts, with the most pronounced effects generally observed at the highest inclusion level. However, this increase in antioxidant capacity was not accompanied by a statistically significant reduction in TBARS values between the experimental variants. Therefore, under the conditions of the present study, the enhanced antioxidant potential of the extracts did not translate into a measurable inhibition of lipid oxidation in the meat matrix. Overall, freeze‐dried *Ribes uva-crispa* can be incorporated into pork pâté at levels of up to 2% without major adverse effects on the physicochemical and textural characteristics evaluated in this study, while increasing the measurable antioxidant potential of the product extracts. Further evaluation of this application should include sensory assessment, as instrumental measurements of color and texture do not fully reflect sensory perception or consumer acceptance, particularly with regard to potential changes in taste, aroma, perceived acidity, and color. In addition, integrating physicochemical and oxidative analyses with microbiological assessment would provide a more comprehensive evaluation of product quality and stability during refrigerated storage. Future studies combining these approaches would allow both the technological suitability and consumer acceptability of pork pâté enriched with freeze‐dried *Ribes uva-crispa* to be more comprehensively established.

## Author Contributions

Conceptualization: Paulina Kęska; methodology: Paulina Kęska and Joanna Stadnik; validation: Paulina Kęska; formal analysis: Paulina Kęska; investigation: Paulina Kęska and Kinga Zmorczyńska; data curation: Paulina Kęska; writing – original draft preparation: Paulina Kęska; writing – review and editing: Joanna Stadnik; visualization: Paulina Kęska; supervision: Joanna Stadnik.

## Funding

No funding was received for this manuscript.

## Disclosure

All authors have read and agreed to the published version of the manuscript.

## Consent

The authors have nothing to report.

## Conflicts of Interest

The authors declare no conflicts of interest.

## Data Availability

The data that support the findings of this study are available from the corresponding author upon reasonable request.
